# Effect of Preserved Versus Preservative‐Free Artificial Tears on the Corneal Epithelial Thickness Mapping by MS‐39 in Dry Eye Patients

**DOI:** 10.1155/joph/9923353

**Published:** 2026-01-08

**Authors:** Ghada A. Nassar, Ahmed Rashad Ashor, Mohamed Hosny, Jorge Alio, Aliaa A. Farag

**Affiliations:** ^1^ Department of Ophthalmology, Faculty of Medicine, Cairo University, Cairo, Egypt, cu.edu.eg; ^2^ Department of Ophthalmology, University Miguel Hernandez, Alicante, Spain, umh.es

**Keywords:** corneal epithelial thickness, dry eye disease, MS-39, preservative-free artificial tears, preserved artificial tears

## Abstract

**Purpose:**

To compare clinical and corneal epithelial changes between preservative and preservative‐free artificial tear therapy using the MS‐39 in patients with dry eye disease (DED).

**Methods:**

This prospective interventional comparative study included 88 eyes of 44 patients. Preserved artificial tears were given for the left eye (group A) and preservative‐free artificial tears for the right eye (group B). They were evaluated before and 3 months after treatment using MS‐39. The dry eye parameters and corneal epithelial thickness were recorded.

**Results:**

There was an increase in the NIBUT and improvement in the severity by OSDI score *(p-value < 0.001)* in each group. There was no significant difference in the mean NIBUT between the two groups *(p-value = 0.470)*. Improvement in the OSDI score *(p-value = 0.026)* and mean post‐treatment epithelial thickness in the central, paracentral superior, and peripheral inferior epithelial areas were significant in the preservative‐free group than the preservative one (*p-value = 0.033, 0.034, and 0.023, respectively*).

**Conclusion:**

Although both preservative‐free and preserved artificial tears show an increase in epithelial thickness using MS‐39 and improvement in OSDI, preservative‐free artificial tears show superiority, subjectively and objectively, compared to preserved artificial tears using even lighter preservatives, being a safer option for long‐term application.

## 1. Introduction

Dry eye disease (DED) is a multifactorial pathology that leads to ocular surface inflammation, tear film instability, and tear hyperosmolarity. Inflammation causes injury to the conjunctival and corneal epithelial cells. Damage to corneal epithelium and nerves may cause symptoms of DED, such as ocular discomfort, dryness, and visual disturbance [1–4].

The diagnosis of DED is based on multiple tests, including the subjective symptoms, visual acuity, tear break‐up time (TBUT), tear osmolarity, tear volume, tear film composition, kerato‐conjunctival vital staining, and ocular biomarkers [5, 6].

Anterior segment optical coherence tomography (AS‐OCT) is a noninvasive imaging method with high‐resolution analysis. AS‐OCT has been preferred in several studies for evaluating corneal epithelium because of its great reliability and repeatability [7, 8].

Owing to the importance of epithelial thickness in the modern evolution of the cornea, different studies have evaluated the epithelial thickness mapping as a valuable marker in the diagnosis, management, and follow‐up of the regenerative process of the corneal epithelial profile in different diseases [9–11].

MS‐39, a unique high‐definition spectral domain AS‐OCT, is a standalone device that uses SD‐OCT and Placido‐disk corneal topography to obtain measurements of the anterior segment of the eye that provides the corneal thickness and epithelial thickness map [12]. Knowledge of the epithelial morphology is useful to assess abnormalities of the ocular surface diseases (OSD) [13, 14].

Artificial tears are used as the first choice in the treatment of DED. They act by promoting corneal epithelial healing, increasing central and peripheral epithelial thickness, and increasing re‐epithelialization of corneal epithelium [15, 16]. However, preservatives may exacerbate ocular inflammation. The most common preservatives are benzalkonium chloride (BAK), chlorobutanol, sodium perborate, thiomersal, and disodium edetate. These preservatives may cause toxic epithelial effect and hypersensitivity reactions, which range from mild irritation to severe corneal scarring [17]. Preservative‐free lubricants could prevent these effects. So, they are indicated for severe dry eye, when higher doses of lubricants are necessary [18].

By reviewing the literature, the effect of preservatives on the corneal epithelial thickness (CET) using MS‐39 in DED has not been fully studied. In our study, we aimed to compare the effect of preserved versus preservative‐free artificial tear therapy on the CET mapping by MS‐39 in patients with DED and correlate the different parameters with visual acuity, symptoms, and signs of DED including noninvasive break‐up time (NIBUT) and Ocular Surface Disease Index (OSDI).

## 2. Materials and Methods

This prospective interventional comparative case series study was performed at the International Femto‐Lasik Center in collaboration with the Ophthalmology Department, Cairo University Hospital, between May 2022 and April 2023. It was approved by the Research Ethics Committee of the Faculty of Medicine, Cairo University, and was conducted in compliance with the principles of the Helsinki Declaration.

All patients signed a written informed consent to participate in the study and for publication of data.

### 2.1. Inclusion Criteria Included

Newly diagnosed as DED.

Patients not receiving any medications for at least 3 months.

Age ranged from 18 to 70 years.

Patients with the NIBUT less than 12 s, and meibomian gland dysfunction (MGD) of different degrees.

### 2.2. Exclusion Criteria Included

Patients with corneal dystrophy, corneal degenerations, or any corneal opacity.

Corneal diseases, such as herpetic keratitis, or OSD, such as ocular cicatricial pemphigoid.

Contact lens wearers.

Keratoconus patients.

Chronic use of preservative‐containing eye drops.

Eyelid disorders.

Previous surgeries especially refractive, ptosis, or eyelid surgery.

Any media opacity, which could prevent high‐quality imaging.

History of diabetes, uveitis, or glaucoma.

Eighty‐eight eyes of 44 patients with DED of various levels of severity. All patients were newly diagnosed as DED. Both eyes of the same patient were compared, and the eyes were divided into two groups.

• Group A: 44 eyes (left eye) receiving preservative artificial tears (hydroxypropyl methylcellulose 3 mg preserved with sodium perborate tetrahydrate—Tears Guard, Orchidia Pharma, Cairo, Egypt) in multidose bottles 5 times daily.
• Group B: 44 eyes (right eye) receiving preservative‐free artificial tears (hypromellose 1.6 mg/0.5 mL—Optilubric, EVA Pharma, Cairo, Egypt) in single‐dose units 5 times daily.

#### 2.2.1. Pretreatment Evaluation

All included patients underwent full ophthalmological examination, including best‐corrected visual acuity (BCVA) in decimal, anterior segment examination, intraocular pressure (IOP) by Goldmann applanation tonometry, NIBUT, and eyelid examination for MGD.

The OSDI score was calculated by the following formula:

OSDI = [(sum of scores for all questions answered) x 100]/[(total number of questions answered) x 4]. The higher the score, the greater the disability.

Based on their OSDI scores, patients can be categorized as having a normal ocular surface (0–12 points) or as having mild (13–22 points), moderate (23–32 points), or severe (33–100 points) OSD [19].

All patients were instructed on how to use topical eye drops especially preservative‐free drops to avoid contamination and to avoid mixing the eye drops between both eyes.

#### 2.2.2. Measurement of CET Mapping by MS‐39 Imaging

**Figure 1:**
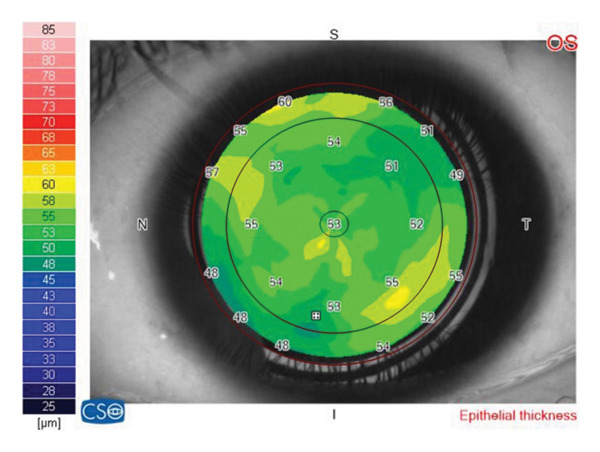
The corneal epithelial thickness map divided into 3 zones: Zone (1) central 2 mm; Zone (2) midperipheral 2–5 mm; and Zone (3) peripheral 5–6 mm. The midperipheral and peripheral zones included the superior, inferior, nasal, and temporal thicknesses.

The CET was evaluated using MS‐39 (Costruzione Strumenti Oftalmici, CSO, Florence, Italy). The acquisition of the images and interpretation of all data of the tests were performed by the primary investigator (G.A.) to avoid interindividual variability. The machine showed excellent repeatability (95%) in measuring the epithelial thickness in keratoconic patients in previous studies [12]. The CET map was recorded and divided into three zones: Zone (1) central 2 mm; Zone (2) midperipheral 2–5 mm; and Zone (3) peripheral 5–6 mm. The midperipheral and peripheral zones included the superior, inferior, nasal, and temporal thicknesses (Figure 1). The pattern of distribution of the epithelial thickness map was documented. Central corneal thickness (CCT) was evaluated. The insufficient quality images detected were repeated.

All patients were examined after 3 months for evaluation of BCVA, refraction, IOP, NIBUT, MGD, and OSDI score. Epithelial mapping and CCT were recorded. The two main groups were compared with analysis of all clinical and investigative data.

Correlations between the different CET parameters, BCVA, NIBUT, and OSDI score were evaluated.

### 2.3. Statistical Analysis

Data were coded and entered using the Statistical Package for the Social Sciences (SPSS) Version 28 (IBM Corp., Armonk, NY, USA). Data were summarized using mean and standard deviation in quantitative data and using frequency (count) and relative frequency (percentage) for categorical data. The continuous variables were checked for normality, and the variables following a normal distribution were analyzed using paired‐sample *t*‐test and Pearson’s correlation coefficient. Non‐normally distributed variables were analyzed with the equivalent nonparametric test (Wilcoxon signed‐rank test and Spearman’s correlation coefficient). Categorical variables were compared using McNemar’s test. *p*‐values less than 0.05 were considered statistically significant.

### 2.4. Sample Size Calculation

A statistical power analysis was performed to compare mean difference for the continuous variables between the two groups using *t*‐test. The effect size in this study was considered to be small to moderate (effect size = 0.35), with an alpha = 0.05 and power = 90%, and the sample size needed with this effect size calculated by G∗ Power 3.1 is 88 eyes (44 eyes for each group) [20].

## 3. Results

### 3.1. Epidemiology and Clinical Data

The mean age was 45.3 ± 11.7 years (range: 18–70). The study included 36 females (81.8%) and 8 males (18.2%). The pretreatment BCVA was 0.72 ± 0.1 in group A versus 0.77 ± 0.1 in group B *(p-value = 0.174)*. The post‐treatment BCVA in group A was 0.78 ± 0.17 and 0.81 ± 0.14 for group B *(p-value = 0.022)*. The mean IOP was 15.12 ± 1.83 mmHg in group A versus 15.21 ± 1.92 mmHg in group B *(p-value = 0.742).* The mean duration of DED was 2.1 ± 2.3 years (range: 0.2–8).

### 3.2. The Pretreatment Data of the Preservative Versus Preservative‐Free Group

No statistically significant difference was found in the mean NIBUT and OSDI scores between groups A and B *(p-value = 0.107 and 0.323, respectively).* The severity by OSDI score in both groups included 11 (25%) mild, 23 (52.3%) moderate, and 10 (22.7%) severe. Group A included 11.4% of patients without MGD and 88.6% with MGD, while in group B, 9.1% of patients did not have MGD and 90.9% had MGD *(p-value = 0.999)*.

However, there was statistically significant difference in the mean epithelial thickness in the peripheral superior and peripheral nasal areas between groups A and B *(p-value = 0.005 and 0.011, respectively*). (Table 1).

**Table 1:** Comparison of the pretreatment data of preservative (group A) versus preservative‐free (group B).

Parameters	Group A	Group B	Difference (left minus right)	*p*‐value
OSDI	50.12 ± 17.73	50.07 ± 17.72	0.05 ± 0.31	0.323
BUT/sec	10.91 ± 2.89	11.67 ± 2.9	−0.77 ± 2.7	0.107
K1	43.35 ± 1.77	43.64 ± 1.45	−0.29 ± 1.38	0.364
K2	44.22 ± 1.85	44.43 ± 1.53	−0.2 ± 1.32	0.666
CCT	532.35 ± 35.45	531.4 ± 29.28	0.95 ± 19.4	0.749
Anterior elevation	9.98 ± 4.13	9.95 ± 4.57	0.02 ± 3.53	0.897
Posterior elevation	−19.91 ± 5.89	−18.95 ± 6.58	−0.95 ± 5.39	0.234
Central	52.58 ± 3.27	52.67 ± 3.62	−0.09 ± 2.85	0.630
Mean paracentral	51.15 ± 3.21	50.56 ± 3.35	0.59 ± 2.43	0.120
Paracentral superior	48.91 ± 3.49	48.05 ± 4.04	0.86 ± 4.58	0.224
Paracentral inferior	52.53 ± 5.03	52.28 ± 4.22	0.26 ± 3.86	0.666
Paracentral nasal	51.65 ± 3.65	51.3 ± 3.97	0.35 ± 3.24	0.485
Paracentral temporal	51.51 ± 3.79	50.63 ± 4.34	0.88 ± 3.74	0.128
Peripheral superior	45.63 ± 4.95	43.6 ± 5.82	2.02 ± 5.04^∗^	0.005^∗^
Peripheral inferior	49.65 ± 5.43	48.53 ± 4.82	1.12 ± 5.43	0.340
Peripheral nasal	51.81 ± 4.33	49.91 ± 4.91	1.91 ± 4.67^∗^	0.011^∗^
Peripheral temporal	49.53 ± 4.01	48.49 ± 4.67	1.05 ± 5.09	0.185

*Note:* K1 flatter corneal meridian, K2 steeper corneal meridian.

Abbreviations: BUT, break‐up time; CCT, central corneal thickness; OSDI, Ocular Surface Disease Index.

^∗^Significant *p*‐value.

### 3.3. The Post‐treatment Data of the Preservative Versus Preservative‐Free Group

There was no statistical significant difference in the mean NIBUT between the two groups *(p-value = 0.470)*. Statistically significant improvement in the OSDI score between the two groups was observed *(p-value = 0.026)*. The severity by OSDI score in both groups included 33 (75%) mild and 11 (25%) moderate.

The mean post‐treatment epithelial thickness in the central, paracentral superior, and peripheral inferior epithelial areas was significantly improved in the preservative‐free group than the preservative one *(p-value = 0.033, 0.034, and 0.023, respectively)* (Table 2).

**Table 2:** Comparison of the post‐treatment data of preservative (group A) versus preservative‐free (group B).

Parameters	Group A	Group B	Difference (left minus right)	*p*‐value
OSDI	31.58 ± 13.22	30.76 ± 12.63	0.82 ± 2.34^∗^	0.026^∗^
BUT/sec	12.88 ± 2.91	13 ± 2.91	−0.12 ± 2.01	0.470
K1	43.49 ± 1.65	43.68 ± 1.42	−0.19 ± 1.23	0.88
K2	44.43 ± 1.75	44.5 ± 1.5	−0.07 ± 1.14	0.690
CCT	533.95 ± 35.97	534.65 ± 30.99	−0.7 ± 20.18	0.822
Anterior elevation	9.7 ± 3.99	9.91 ± 4.13	−0.21 ± 3.87	0.651
Posterior elevation	−18.95 ± 5.32	−18.7 ± 6.72	−0.26 ± 5.56	0.913
Central	53.77 ± 3.77	54.79 ± 4.22	−1.02 ± 3.05^∗^	0.033^∗^
Mean paracentral	52.32 ± 3.65	53.11 ± 3.29	−0.78 ± 2.61	0.056
Paracentral superior	49.3 ± 3.54	50.58 ± 4.04	−1.28 ± 3.83^∗^	0.034^∗^
Paracentral inferior	53.98 ± 5.04	54.53 ± 4.19	−0.56 ± 3.76	0.336
Paracentral nasal	53.47 ± 4.43	54.26 ± 4.25	−0.79 ± 3.78	0.178
Paracentral temporal	52.56 ± 4.33	53.07 ± 4.03	−0.51 ± 4.65	0.475
Peripheral superior	47.28 ± 5.76	47.42 ± 6.15	−0.14 ± 6.23	0.990
Peripheral inferior	50.37 ± 5.12	51.95 ± 5.15	−1.58 ± 5.32^∗^	0.023^∗^
Peripheral nasal	53.58 ± 4.72	53.49 ± 5.52	0.09 ± 4.97	0.604
Peripheral temporal	51.09 ± 4.09	51.67 ± 4.68	−0.58 ± 5.06	0.456

*Note:* K1 flatter corneal meridian, K2 steeper corneal meridian.

Abbreviations: BUT, break‐up time; CCT, central corneal thickness; OSDI, Ocular Surface Disease Index.

^∗^Significant *p*‐value.

### 3.4. Comparison of Pretreatment and Post‐treatment Data in the Preservative Group

By further analysis of data in group A, there was statistically significant increase in the NIBUT with decrease in the OSDI score and improvement in the severity by OSDI score *(p-value < 0.001 each).*

**Figure 2:**
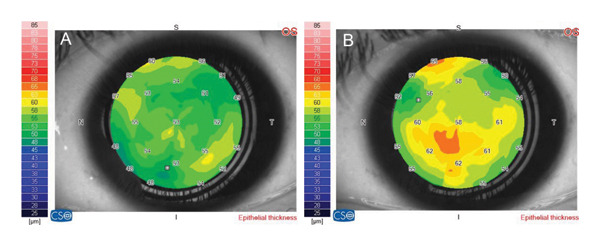
(A) Corneal epithelial thickness map before treatment of case no. 11 in group A (preservative group). (B) Corneal epithelial thickness map 3 months after treatment of the same patient in group A.

In the corneal epithelial map parameters, there was significant increase in the central epithelial area *(p-value < 0.001),* the paracentral inferior, nasal, and temporal areas *(p-value = 0.002, < 0.001, 0.018)*, respectively, and the peripheral superior, nasal, and temporal areas *(p-value = 0.006, 0.001, < 0.001)*, respectively (Table 3) (Figure 2 A & B).

**Table 3:** Comparison of different parameters between the pretreatment and post‐treatment follow‐up in the preservative (group A).

Parameters	Pretreatment	Post‐treatment	Difference (pre‐ minus post‐treatment)	*p*‐value
OSDI	50.12 ± 17.73	31.58 ± 13.22	18.54 ± 14.71^∗^	< 0.001^∗^
BUT/sec	10.91 ± 2.89	12.88 ± 2.91	−1.98 ± 2.39^∗^	< 0.001^∗^
K1	43.35 ± 1.77	43.49 ± 1.65	−0.14 ± 0.49	0.175
K2	44.22 ± 1.85	44.43 ± 1.75	−0.21 ± 0.54^∗^	< 0.001^∗^
CCT	532.35 ± 35.45	533.95 ± 35.97	−1.61 ± 3.8^∗^	0.008^∗^
Anterior elevation	9.98 ± 4.13	9.7 ± 3.99	0.28 ± 1.86	0.330
Posterior elevation	−19.91 ± 5.89	−18.95 ± 5.32	−0.95 ± 2.87^∗^	0.035^∗^
Central	52.58 ± 3.28	53.77 ± 3.77	−1.19 ± 1.64^∗^	< 0.001^∗^
Mean paracentral	51.15 ± 3.21	52.33 ± 3.65	−1.17 ± 1.84^∗^	< 0.001^∗^
Paracentral superior	48.91 ± 3.49	49.3 ± 3.54	−0.4 ± 2.94	0.382
Paracentral inferior	52.53 ± 5.03	53.98 ± 5.04	−1.44 ± 2.79^∗^	0.002^∗^
Paracentral nasal	51.65 ± 3.65	53.47 ± 4.43	−1.81 ± 2.25^∗^	< 0.001^∗^
Paracentral temporal	51.51 ± 3.79	52.56 ± 4.33	−1.05 ± 2.78^∗^	0.018^∗^
Peripheral superior	45.63 ± 4.95	47.28 ± 5.76	−1.65 ± 3.86^∗^	0.006^∗^
Peripheral inferior	49.65 ± 5.43	50.37 ± 5.12	−0.72 ± 3.02	0.096
Peripheral nasal	51.81 ± 4.33	53.58 ± 4.72	−1.77 ± 3.41^∗^	0.001^∗^
Peripheral temporal	49.53 ± 4.01	51.09 ± 4.09	−1.56 ± 2.5^∗^	< 0.001^∗^

*Note:* K1 flatter corneal meridian, K2 steeper corneal meridian.

Abbreviations: BUT, break‐up time; CCT, central corneal thickness; OSDI, Ocular Surface Disease Index.

^∗^Significant *p*‐value.

### 3.5. Comparison of Pretreatment and Post‐treatment Data in the Preservative‐Free Group

In group B, there was statistically significant increase in the NIBUT, decrease in the OSDI score, and improvement of the severity of the OSDI score *(p-value < 0.001 each).* However, the difference in the MGD was statistically nonsignificant *(p-value = 0.500)*.

**Figure 3:**
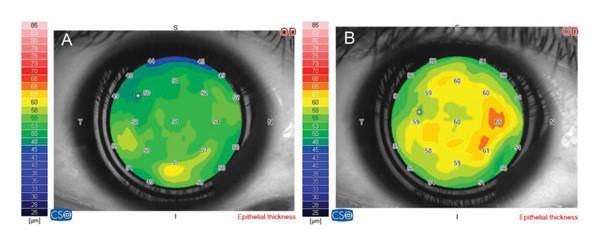
(A) Corneal epithelial thickness map before treatment of case no. 20 in group B (preservative‐free group). (B) Corneal epithelial thickness map 3 months after treatment of the same patient in group B.

In the corneal epithelial parameters, there was significant increase in all parameters of the epithelial map: the central epithelial area; the paracentral superior, inferior, nasal, and temporal areas; and the peripheral superior, inferior, nasal, and temporal areas *(p-value < 0.001 each)* (Table 4) (Figure 3 A & B).

**Table 4:** Comparison of different parameters between the pretreatment and post‐treatment follow‐up in the preservative‐free (group B).

Parameters	Pretreatment	Post‐treatment	Difference (pre‐ minus post‐treatment)	*p*‐value
OSDI	50.07 ± 17.72	30.76 ± 12.63	19.32 ± 14.76^∗^	< 0.001^∗^
BUT/sec	11.67 ± 2.9	13 ± 2.91	−1.33 ± 2.18^∗^	< 0.001^∗^
K1	43.64 ± 1.45	43.68 ± 1.42	−0.04 ± 0.32	0.270
K2	44.43 ± 1.53	44.5 ± 1.5	−0.07 ± 0.31	0.224
CCT	531.4 ± 29.28	534.65 ± 31	−3.26 ± 6.87^∗^	0.003^∗^
Anterior elevation	9.95 ± 4.57	9.91 ± 4.13	0.05 ± 2.09	0.896
Posterior elevation	−18.95 ± 6.58	−18.7 ± 6.72	−0.26 ± 2.88	0.429
Central	52.67 ± 3.62	54.79 ± 4.22	−2.12 ± 2.11^∗^	< 0.001^∗^
Mean paracentral	50.56 ± 3.35	53.11 ± 3.29	−2.55 ± 2.23^∗^	< 0.001^∗^
Paracentral superior	48.05 ± 4.04	50.58 ± 4.04	−2.54 ± 3.57^∗^	< 0.001^∗^
Paracentral inferior	52.28 ± 4.22	54.53 ± 4.19	−2.26 ± 2.36^∗^	< 0.001^∗^
Paracentral nasal	51.3 ± 3.97	54.26 ± 4.25	−2.95 ± 3.00^∗^	< 0.001^∗^
Paracentral temporal	50.63 ± 4.34	53.07 ± 4.03	−2.44 ± 3.03^∗^	< 0.001^∗^
Peripheral superior	43.6 ± 5.82	47.42 ± 6.15	−3.81 ± 3.16^∗^	< 0.001^∗^
Peripheral inferior	48.53 ± 4.82	51.95 ± 5.15	−3.42 ± 4.51^∗^	< 0.001^∗^
Peripheral nasal	49.91 ± 4.91	53.49 ± 5.52	−3.58 ± 3.64^∗^	< 0.001^∗^
Peripheral temporal	48.49 ± 4.67	51.67 ± 4.68	−3.19 ± 3.63^∗^	< 0.001^∗^

*Note:* K1 flatter corneal meridian, K2 steeper corneal meridian.

Abbreviations: BUT, break‐up time; CCT, central corneal thickness; OSDI, Ocular Surface Disease Index.

^∗^Significant *p*‐value.

A statistically significant positive correlation was found between the percentage improvement of OSDI score and post‐treatment increase in epithelial thickness in the peripheral nasal area in group A *(r = 0.337, p = 0.027)*. This was also observed in the central and paracentral superior areas in group B *(r = 0.327, p = 0.032, r = 0.314, p = 0.040)*, respectively (Table 5).

**Table 5:** Correlation between the percentage change of OSDI score with the post‐treatment epithelial thickness in both groups A and B.

		Group A	Group B
Central	*r*	0.218	0.327
	*p*‐value	0.159	0.032^∗^
Paracentral superior	*r*	0.082	0.314
	*p*‐value	0.599	0.040^∗^
Paracentral inferior	*r*	−0.092	0.080
	*p*‐value	0.556	0.608
Paracentral nasal	*r*	0.173	0.186
	*p*‐value	0.269	0.233
Paracentral temporal	*r*	0.134	0.099
	*p*‐value	0.390	0.526
Peripheral superior	*r*	0.177	0.170
	*p*‐value	0.255	0.276
Peripheral inferior	*r*	−0.025	0.019
	*p*‐value	0.876	0.902
Peripheral nasal	*r*	0.337^∗^	0.058
	*p*‐value	0.027	0.711
Peripheral temporal	*r*	−0.026	−0.076
	*p*‐value	0.867	0.626

*Note:* *r* correlation coefficient.

^∗^Significant *p* value.

## 4. Discussion

The use of artificial tears for the treatment of dry eye is widespread especially where aqueous‐deficient DED exists [21].

Drops delivered in a multidose format have preservatives for maintaining their sterility. The most commonly used preservative is BAK. While it is known to be an effective antimicrobial agent, many studies have shown its adverse effect on the ocular surface particularly when used for a long time. This has contributed to a movement toward preservative‐free topical preparations in both glaucomatous and DED populations [21].

The corneal epithelium is one of the main structures that undergo degenerative alterations in ocular diseases. Therefore, CET measurement has been suggested as an objective indicator for monitoring ocular surface health [21].

The effect of preservatives on epithelial cells has been the interest of many studies [20, 21]. The tear film proteome, for factors relating to epithelial leakage and inflammation, showed significant improvement after switching to preservative‐free tafluprost from BAK‐preserved latanoprost drops [21].

Experimental studies on corneal epithelial cells showed that the preservative‐free anti‐allergy drug ketotifen did not impair cell structure and viability, compared to the damage resulting from use of BAK‐preserved medications [21–23]. Following 7‐day treatment with preservative‐free travoprost, positive effects on tear production and corneal tissues were compared to the BAK‐treated group [21]. Similar conclusions were reached using scanning electron microscopy to examine rabbit eyes [21].

The toxic effect of BAK lies in loss of epithelial microvillar brush, widening of the extracellular space, and disorganization of the epithelium. This results in a decrease in the conjunctival and corneal epithelium cell density and metaplasia through apoptosis and/or cellular necrosis, depending on the concentration [24].

Controversies exist with the effect of dry eye on the corneal morphology [25], and some animal and human studies found that the CET became thicker in DED, indicating that epithelial proliferation has a significant impact on the inflammatory process [22]. Others reported that the CET tends to be thinner in dry eyes and attributed it to the destruction of stem cells at the limbus [26].

Nam & kim were the first to investigate the effect of preservative including BAK on the CET using OCT and concluded that there was a linear relationship between the increased number of medications, treatment duration, and preservatives contributed to reduced epithelial thickness [27].

To our knowledge, this is the first study using MS‐39 to evaluate the epithelial thickness in dry eye patients and the effect of preservative and preservative‐free lubricants on the corneal epithelium [28]. Although in our study we did not compare the epithelial thickness with the normal population, the significant increase in thickness post‐treatment in both groups denotes the presence of pre‐existing thinning in the corneal epithelium which may be attributed to the decrease in the inflammatory process and promotion of proliferation of the epithelial cells especially at the limbus, with the added benefit of less induced inflammatory reaction of the added preservative.

Cost, ease of use, and compliance remain relevant considerations for preservative‐free single‐use formats. For mild‐to‐moderate DED, alternative artificial tear and lubricants exist with preservatives other than BAK [21].

A number of in vitro studies have been conducted comparing the effect of BAK, sodium perborate, other alternative preservatives, and preservative‐free formulations on human conjunctival epithelial cells. Across these studies, the general conclusion is that sodium perborate has a significantly less toxic effect on ocular corneal and conjunctival cells compared to BAK but that preservative‐free preparations have the least effect on cell viability [29, 30].

In our study, we aimed to compare the effect of lighter preservatives as sodium perborate to preservative‐free preparations. The idea of a “disappearing” preservative is to deliver both antimicrobial activities in the solution and then cause minimal impact to the ocular surface [31].

Previous switch studies have shown that even with non‐BAK preservatives, switching to preservative‐free resulted in quick improvement of OSDI and punctate keratitis [32–34]. However, the tests used as OSDI and TBUT lack standardization and repeatability with great discrepancy between the symptoms and signs in dry eye patients [35].

To provide better reproducibility, we used the NIBUT with cutoff point of 12 s which was proven to be the value with greatest sensitivity and specificity, distinguishing patients with dry eye symptoms and normal subjects. NIBUT has shown better diagnostic performance than standard TBUT in detecting patients with dry eye symptoms [36]. In our study, both preservative and preservative‐free artificial tears had significant improvement of the NIBUT.

In addition to the improvement in the OSDI and NIBUT in our study, superiority of the preservative‐free medication was detected using the OCT with significant changes regarding central, paracentral superior, and paracentral inferior areas.

Cui et al. demonstrated greater variability in epithelial thickness in the superior quadrant in patients with dry eye, which would explain, in our study, the pretreatment significant difference in the superior corneas of both groups, which can also be due to the variability of pannus between both eyes [25].

Vision was also statistically improved in the preservative‐free group. Studies have found that the visual disturbances in DED are related to the unstable tear film and have been considered as a parameter for evaluation of the stability of the tear film and the severity of DED [37].

The main strength of our study is the combination of both the subjective and objective measures of DED, as well as the association of clinical parameters of DED and epithelial thickness mapping, which all proved to be safer in the preservative‐free group.

Limitations include short follow‐up period, which might have not demonstrated clearly the toxic effect of the preservative. Another limitation is using only NITBUT as an objective tool, and other tests, such as Schirmer’s test, fluorescein staining, and quantitative measurement of tear film osmolarity, should be added in further studies. Because of the low socioeconomic lifestyle, some of the questions of the OSDI did not apply as using the TV, driving, and air conditioning might have been misleading regarding the severity of their condition.

The presence of treatment discrepancies and the peripheral nasal and peripheral superior quadrants between both groups are a potential limitation; however, this is attributed to the endemic trachoma with unequal pannus between both eyes in some patients, and discrepancies are only seen. In the very peripheral cornea, however, the central and paracentral quadrants that are more relevant to the study are comparable between both groups.

Another limitation is the lack of normal control groups. Further, case–control studies are needed to compare the effect of artificial tears on non‐DED patients versus those with pre‐existing DED.

In conclusion, although both preservative‐free and preserved artificial tears show an increase in epithelial thickness using MS‐39 and improvement in OSDI. Preservative‐free artificial tears show superiority, both subjectively and objectively, compared to preserved artificial tears using even lighter preservatives, being a safer option for long‐term application.

## Ethics Statement

The Institutional Review Board and Ethics Committee of the Faculty of Medicine, Cairo University, approved this study. Ethics Committee Code N‐12‐2022.

## Disclosure

All authors read and approved the final manuscript.

## Conflicts of Interest

The authors declare no conflicts of interest.

## Author Contributions

Conceptualization: Ghada A. Nassar, Aliaa A. Farag, Ahmed Rashad Ashor, and Mohamed Hosny; methodology and formal analysis: Ghada A. Nassar, Aliaa A. Farag, and Ahmed Rashad Ashor; writing–original draft preparation: Ghada A. Nassar, Aliaa A. Farag, and Ahmed Rashad Ashor; writing–review and editing: Mohamed Hosny and Jorge Alio; supervision: Mohamed Hosny and Jorge Alio.

## Funding

The authors received no financial support.

## Data Availability

All data used and/or analyzed during this study are available and can be presented by the corresponding author upon a reasonable request.

## References

[1] Craig J. P. , Nichols K. K. , Akpek E. K. et al., TFOS DEWS II Definition and Classification Report, Ocular Surface. (2017) 15, no. 3, 276–283, 10.1016/j.jtos.2017.05.008, 2-s2.0-85025470412. 28736335

[2] Sledge S. M. , Khimji H. , Borchman D. et al., Evaporation and Hydrocarbon Chain Conformation of Surface Lipid Films, Ocular Surface. (2016) 14, no. 4, 447–459, 10.1016/j.jtos.2016.06.002, 2-s2.0-84992390303. 27395776 PMC5065757

[3] Asiedu K. , Role of Ocular Surface Neurobiology in Neuronal-mediated Inflammation in Dry Eye Disease, Neuropeptides. (2022) 95, 10.1016/j.npep.102266. 35728484

[4] Belmonte C. , Nichols J. J. , Cox S. M. et al., TFOS DEWS II Pain and Sensation Report, Ocular Surface. (2017) 15, no. 3, 404–437, 10.1016/j.jtos.2017.05.002, 2-s2.0-85025444293. 28736339 PMC5706540

[5] Inomata T. , Shiang T. , Iwagama M. et al., Changes in Distribution of Dry Eye Disease by the New 2016 Diagnostic Criteria from the Asia Dry Eye Society, Scientific Reports. (2018) 8, no. 1, 10.1038/s41598-018-19775-3, 2-s2.0-85041237241. PMC5789837 29382858

[6] Yokoi N. , Georgiev G. A. , Kato H. et al., Classification of Fluorescein Breakup Patterns: A Novel Method of Differential Diagnosis for Dry Eye, American Journal of Ophthalmology. (2017) 180, 72–85, 10.1016/j.ajo.2017.05.022, 2-s2.0-85020861658. 28579061

[7] Gaudenzi D. , Mori T. , Crugliano S. et al., AS-OCT and Ocular Hygrometer as Innovative Tools in Dry Eye Disease Diagnosis, Applied Sciences. (2022) 12, no. 3, 10.3390/app12031647.

[8] Akiyama R. , Usui T. , and Yamagami S. , Diagnosis of Dry Eye by Tear Meniscus Measurements Using Anterior Segment Swept Source Optical Coherence Tomography, Cornea. (2015) 34, no. 11, 115–120, 10.1097/ico.0000000000000583, 2-s2.0-84944046264. 26448168

[9] Loureiro T. , Rodrigues-Barros S. , Carreira A. R. et al., Corneal Epithelial Thickness Changes After Topical Treatment of Dry Eye Disease in Primary Sjögren Syndrome, Clinical Ophthalmology. (2023) 17, 993–1005, 10.2147/opth.s375505. 37035513 PMC10075387

[10] Khamar P. , Wadia K. , Dalal R. , Grover T. , Versaci F. , and Gupta K. , Advanced Epithelial Mapping for Refractive Surgery, Indian Journal of Ophthalmology. (2020) 68, no. 12, 2819–2830, 10.4103/ijo.ijo_2399_20. 33229657 PMC7856960

[11] Kim K. , Lee S. , Jeon Y. , and Min J. , Anterior Segment Characteristics in Normal and Keratoconus Eyes Evaluated with a New Type of swept-source Optical Coherence Tomography, PLoS One. (2022) 1, no. 9, 10.1371/journal.pone.0274071. PMC9436129 36048835

[12] Vega-Estrada A. , Mimouni M. , Espla E. , Barrio J. , and Alio J. , Corneal Epithelial Thickness Intra-subject Repeatability and its Relation with Visual Limitation in Keratoconus, American Journal of Ophthalmology. (2019) 200, 255–262, 10.1016/j.ajo.2019.01.015, 2-s2.0-85062024164. 30689987

[13] Ma J. X. , Wang L. , Weikert M. P. , Montes I. , and Koch D. , Evaluation of the Repeatability and Reproducibility of Corneal Epithelial Thickness Mapping for a 9-mm Zone Using Optical Coherence Tomography, Cornea. (2019) 38, no. 1, 67–73, 10.1097/ico.0000000000001806, 2-s2.0-85060129626. 30379719

[14] Georgeon C. , Marciano I. , Cuyaubère R. , Sandali O. , Bouheraoua N. , and Borderie V. , Corneal and Epithelial Thickness Mapping: Comparison of Enhanced Spectral-Domain- and Spectral-domain-optical Coherence Tomography, Journal of Ophthalmology. (2021) 2021, 1–6, 10.1155/2021/3444083. PMC8510821 34650817

[15] Cakir B. , Dogan E. , Celik E. , Babashli T. , Ucak T. , and Alagoz G. , Effects of Artificial Tear Treatment on Corneal Epithelial Thickness and Corneal Topography Findings in Dry Eye Patients, Journal Français d′Ophtalmologie. (2018) 41, no. 5, 407–411, 10.1016/j.jfo.2017.06.032, 2-s2.0-85047084706. 29776765

[16] Eric C. , Winston Y. , and Abayomi O. , Impact of Hyaluronic Acid-containing Artificial Tear Products on Re-epithelialization in an in-vivo Corneal Wound Model, Journal of Ocular Pharmacology and Therapeutics. (2018) 34, no. 4, 360–364, 10.1089/jop.2017.0080, 2-s2.0-85046904493. 29394128 PMC5952336

[17] Jee D. , Park S. H. , Kim M. S. , and Kim E. C. , Antioxidant and Inflammatory Cytokine in Tears of Patients with Dry Eye Syndrome Treated with Preservative Free Versus Preserved Eye Drops, Investigative Ophthalmology & Visual Science. (2014) 55, no. 8, 5081–5089, 10.1167/iovs.14-14483, 2-s2.0-84906057384. 24994869

[18] Inoue D. , Mohamed Y. H. , Uematsu M. , and Kitaoka T. , Corneal Damage and its Recovery After Instillation of Preservative-free Versus Preserved Latanoprost Eye Drops, Cutan Ocul Toxicol Jun. (2020) 39, no. 2, 158–164, 10.1080/15569527.2020.1752228. 32295438

[19] Miller K. L. , Walt J. G. , Mink D. R. et al., Minimal Clinically Important Difference for the Ocular Surface Disease Index, Archives of Ophthalmology. (2010) 128, no. 1, 94–101, 10.1001/archophthalmol.2009.356, 2-s2.0-75349101861. 20065224

[20] Faul F. , Erdfelder E. , Lang A. , and Buchner A. , G∗ Power 3: A Flexible Statistical Power Analysis Program for the Social, Behavioral and Biomedical Sciences, Behavior Research Methods. (2007) 39, no. 2, 175–191, 10.3758/bf03193146, 2-s2.0-34547145165. 17695343

[21] Walsh K. and Jones L. , The Use of Preservatives in Dry Eye Drops, Clinical Ophthalmology. (2019) 13, 1409–1425, 10.2147/OPTH.S211611, 2-s2.0-85070940122. 31447543 PMC6682755

[22] Kanellopoulos A. and Asimellis G. , In Vivo 3-dimensional Corneal Epithelial Thickness Mapping as an Indicator of Dry Eye: Preliminary Clinical Assessment, American Journal of Ophthalmology. (2014) 157, no. 1, 63–68, 10.1016/j.ajo.2013.08.025, 2-s2.0-84890569917. 24200234

[23] Berdy J. , Abelson M. , Smith L. , and George A. , Preservative-Free Artificial Tear Preparations. Assessment of Corneal Epithelial Toxic Effects, Archives of Ophthalmology. (1992) 110, no. 4, 528–532, 10.1001/archopht.1992.01080160106043, 2-s2.0-0026516506. 1562263

[24] Coroi M. C. , Bungau S. , and Tit M. , Preservatives from the Eye Drops and the Ocular Surface, Rom J Ophthalmol. (2015) 59, no. 1, 2–5. 27373107 PMC5729814

[25] Cui X. , Hong J. , Wang F. et al., Assessment of Corneal Epithelial Thickness in Dry Eye Patients, Optometry and Vision Science. Official Publication of the American Academy of Optometry. (2014) 91, no. 12, 1446–1454, 10.1097/OPX.0000000000000417, 2-s2.0-84924758877. 25279779 PMC4302058

[26] Erdélyi B. , Kraak R. , Zhivov A. , Guthoff R. , and Németh J. , In Vivo Confocal Laser Scanning Microscopy of the Cornea in Dry Eye, Graefe’s Archive for Clinical and Experimental Ophthalmology. (2007) 245, 39–44. 10.1007/s00417-006-0375-6 16874525

[27] Nam M. and Kim W. , Changes in Corneal Epithelial Thickness Induced by Topical Antiglaucoma Medications, Journal of Clinical Medicine. (2021) 10, no. 16, 10.3390/jcm10163464. PMC8397015 34441760

[28] Feng Y. , Reinstein D. , Nitter T. et al., Epithelial Thickness Mapping in Keratoconic Corneas: Repeatability and Agreement Between CSO MS-39, Heidelberg Anterion, and Optovue Avanti OCT Devices, Journal of Refractive Surgery. (2023) 39, no. 7, 474–480, 10.3928/1081597x-20230606-01. 37449505

[29] Fabiani C. , Barabino S. , Rashid S. , and Dana R. , Corneal Epithelial Proliferation and Thickness in a Mouse Model of Dry Eye, Experimental Eye Research. (2009) 89, no. 2, 166–171, 10.1016/j.exer.2009.03.003, 2-s2.0-67650627754. 19298814 PMC2713794

[30] Brasnu E. , Brignole-Baudouin F. , Riancho L. , Guenoun M. , Warnet J. , and Baudouin C. , In- Vitro Effects of Preservative-free Tafluprost and Preserved Latanoprost, Travoprost, and Bimatoprost in a Conjunctival Epithelial Cell Line, Current Eye Research. (2008) 33, no. 4, 303–312, 10.1080/02713680801971857, 2-s2.0-41949097556. 18398704

[31] Xu M. , Sivak G. , and McCanna D. , Comparison of the Effects of Ophthalmic Solutions on Human Corneal Epithelial Cells Using Fluorescent Dyes, Journal of Ocular Pharmacology and Therapeutics. (2013) 29, no. 9, 794–802, 10.1089/jop.2013.0002, 2-s2.0-84887072512. 23905770

[32] Iester M. , Telani S. , Frezzotti P. et al., Ocular Surface Changes in Glaucomatous Patients Treated with and Without Preservatives Beta-blockers, Journal of Ocular Pharmacology and Therapeutics. (2014) 30, no. 6, 476–481, 10.1089/jop.2013.0216, 2-s2.0-84903700814. 24787056

[33] Uusitalo H. , Egorov E. , Kaarniranta K. , Astakhov Y. , and Ropo A. , Benefits of Switching from Latanoprost to Preservative-free Tafluprost Eye Drops: A Meta-analysis of Two Phase IIIb Clinical Trials, Clinical Ophthalmology. (2016) 10, 445–454, 10.2147/opth.s91402, 2-s2.0-84961241786. 27041987 PMC4801127

[34] Nasser L. , Rozycka M. , Rendon G. , and Navas A. , Real-Life Results of Switching from Preserved to Preservative Free Artificial Tears Containing Hyaluronate in Patients with Dry Eye Disease, Clinical Ophthalmology. (2018) 12, 1519–1525, 10.2147/opth.s160053, 2-s2.0-85057741088. 30197497 PMC6112802

[35] Abou S. M. , Wang J. , Kontadakis G. et al., Corneal Epithelial Thickness Profile in Dry-eye Disease, Eye. (2020) 34, no. 5, 915–922, 10.1038/s41433-019-0592-y. 31576026 PMC7182579

[36] Vidas P. S. , Petriček I. , Jukić T. et al., Noninvasive Tear Film Break-up Time Assessment Using Handheld Lipid Layer Examination Instrument, Acta Clinica Croatica. (2019) 58, no. 1, 63–71, 10.20471/acc.2019.58.01.09, 2-s2.0-85069297592. 31363327 PMC6629192

[37] Wei Z. , Su Y. , Su G. , Baudouin C. , Labbé A. , and Liang Q. , Effect of Artificial Tears on Dynamic Optical Quality in Patients with Dry Eye Disease, BMC Ophthalmology. (2022) 22, no. 1, 10.1186/s12886-022-02280-7. PMC8830171 35144571

